# School Climate, Absenteeism and School Transfer Due to Transphobic Bullying in Transgender And Non‐Binary Youth

**DOI:** 10.1002/jad.70084

**Published:** 2025-12-15

**Authors:** Anna Martha Vaitses Fontanari, Marina Feijó, Anna Paula Villas‐Boas, Guilherme Welter Wendt, Angelo Brandelli Costa

**Affiliations:** ^1^ Pontificia Universidade Catolica do Rio Grande do Sul/PORTO ALEGRE State of Rio Grande Do Sul Brazil; ^2^ Universidade Estadual do Oeste do Parana ‐ Campus Francisco Beltrao Francisco Beltrao, State of Parana Brazil; ^3^ John Cabot University Rome Lazio Italy

**Keywords:** gender non‐binary youth, school absenteeism, school climate, school transfer, transgender youth, transphobia

## Abstract

**Introduction:**

This study investigates the critical role of school climate in relation to absenteeism and school transfer due to transphobic bullying among transgender and gender non‐binary youth in Brazil, a context where research on this population is scarce. School climate, characterized by feelings of belonging, safety, and positive relationships, has been shown to significantly impact academic outcomes and overall well‐being. Understanding these dynamics is crucial to supporting transgender and gender non‐binary students, who often face unique challenges in educational settings.

**Methods:**

A total of 293 Brazilian transgender and gender non‐binary youth, with a mean age of 18.71 years (ranging from 13 to 25 years) participated in the study. Gender identities comprised 45.02% transgender girls, 22.34% transgender boys, and 32.65% non‐binary individuals, 58.87% White and 41.13% Non‐White. Measures included assessments of school connectedness, school safety, and experiences with transphobic bullying.

**Results:**

High rates of school disruption were prevalent; 45.4% of participants reported prolonged absences of more than two consecutive weeks, and over half (55.6%) had wanted to change schools due to transphobic bullying. Students in post‐high school settings reported statistically significantly higher levels of school connectedness and perceived safety compared to students in high school. However, perceived safety in gender‐segregated spaces, such as washrooms and changing rooms, was critically low for both groups. Furthermore, higher perceived support for gender affirmation from peers and teachers was strongly and positively correlated with increased school connectedness and safety across all contexts.

**Conclusions:**

Low school connectedness and safety are significant risk factors for school disruption among Brazilian transgender and non‐binary youth. The transition from high school to post‐secondary education is associated with an improved school climate, yet critical safety issues in specific areas persist across all educational levels. These findings underscore the urgent need for targeted interventions in Brazilian schools—particularly within secondary education—to foster supportive, gender‐affirming relationships and implement policies.

## 1 Introduction

The relationships between school connectedness and dropout have been particularly salient in transgender youth as this population is relatively more exposed to environmental stressors (Feijo et al. 2022; Valentine and Shipherd 2018). Since social structures are most frequently not designed to accommodate gender diversity, transgender individuals often experience direct and systematic marginalization (Hendricks and Testa 2012). As a result, transgender students often experience discrimination and violence at school, which can lead to school disconnectedness (Day et al. 2018; Fontanari et al. 2018; McGuire et al. 2010a; Zeluf et al. 2016).

School climate alludes to a sense of belonging, positive interpersonal relationships, and feeling safe in the school environment (Cohen 2009). Understanding the role of school climate has become a focus of research interest in the last few years, particularly in the US (Cohen 2009; Thapa and Guffey 2013), with increasing awareness about how systemic factors shape school‐related variables (Hendricks and Testa 2012).

Evidence has shown that a supportive school climate is linked with improved academic outcomes (Hinduja et al. 2024; Maxwell and Maxwell 2016) and lower levels of school absenteeism (Kearney and Hendron 2016). Beyond the influences on one's achievements, school climate is linked to adolescent health and well‐being (Modin and Östberg 2009; Saab and Klinger 2010).

School connectedness, as measured by inquiring about the presence of caring relationships with adults and peers at school (Centers for Disease Control and Prevention 2009), has been associated with higher academic achievement and better mental health outcomes, such as less frequent suicidal ideation among Lesbian, Gay, Bisexual and Transgender (LGBT) youth (Whitaker et al. 2016). Research aiming to evaluate school climate usually applies safety and school connectedness measurements (Maxwell and Maxwell 2016; Thapa and Guffey 2013).

Although improving remarkably since the nineties, school enrollment and dropout rates are still worse in Latin America when compared to developed countries, especially considering gender minorities and lower socioeconomic status (SES; Bassi et al. 2013). Nonetheless, existing studies from Latin America have found associations between school climate and well‐being (Varela et al. 2019), prosocial behaviors (Kanacri et al. 2017), and school performance (López et al. 2017).

In the US, most transgender students report feeling unsafe at school because of their gender identity (Greytak et al. 2009; Greytak et al. 2016; Grossman et al. 2009; McGuire et al. 2010a). This is supported by the evidence on US transgender students experiencing high levels of school victimization, varying from verbal harassment to physical assault (Day et al. 2018; Grossman et al. 2009; McGuire et al. 2010b). Compared to their cisgender peers, transgender students from the US were more likely to have been bullied at school and have been absent from school for three or more days in a month (Pampati et al. 2018).

In addition, almost half of lesbian, gay, bisexual, transgender, and queer or questioning (LGBTQ+) students feel unsafe at school because of their minority status (Kosciw et al. 2018). Consequently, one in ten transgender youth students missed four or more days of school in a month due to feeling uncomfortable or unsafe (Kosciw et al. 2018). Participants also reported avoiding gender‐segregated spaces in schools, such as bathrooms and locker rooms, for fear of harassment (Kosciw et al. 2018).

Hostile school environment can negatively affect the academic success and, later, the labor market engagement of transgender youth (Birkett et al. 2009; Kosciw et al. 2016). Relatedly, educational attainment has been linked to later success in terms of income, occupational status, and life satisfaction (Mirowsky and Ross 1998; Ross and Wu 1995). Indeed, US transgender youth who experienced harassment were more likely to miss school due to safety concerns than their cisgender peers (Greytak et al. 2016). Furthermore, transgender students had lower grades and were less ambitious when it came to college attendance (Kosciw et al. 2016).

Due to critical socio‐cultural differences, findings from studies from developed countries cannot be generalized to other nations. One example is that most studies investigating school climate do not consider SES and social support as potentially confounding the relationship between school climate and student outcomes (Sellstrom and Bremberg 2006). Indeed, lower SES is widespread in developing countries and has been associated with school climate variables, including fewer extracurricular activities (Greytak et al. 2016). Moreover, SES has repeatedly been linked with elevated dropout rates (Cairns et al. 1989; Cholewa et al. 2017; Gonzalez‐Betancor and Lopez‐Puig 2016; Lansford et al. 2017; Ross and Wu 1995; Wood et al. 2017), equally moderating the relationship between school climate and physical abuse (Jain et al. 2018). A known protective factor against adversities among adolescents is social support, which sustains the claim that both SES and social support should be considered as potentially affecting the relationships between school connectedness and dropout (Marie et al. 2018; Valentine and Shipherd 2018; Veale et al. 2017).

In Brazil, transgender and non‐binary youth face pervasive challenges within educational settings, including high rates of verbal and physical victimization and systematic exclusion from school spaces. National survey data indicate that 43% of LGBTQ+ students feel unsafe at school due to their gender identity, and more than half report exposure to derogatory comments about being transgender (Associação Brasileira de Lésbicas, Gays, Bissexuais, Travestis e Transexuais ‐ ABGLT 2016). These experiences often lead to avoidance of gender‐segregated facilities, such as bathrooms and locker rooms, and are associated with increased absenteeism and psychological distress (Bento 2011; Cruz 2011; de Souza and Bernardo 2014). Despite these alarming patterns, quantitative research on school climate for transgender youth in Latin America remains scarce, underscoring the need for studies that examine how institutional factors—such as safety, connectedness, and gender‐affirmation practices—shape educational outcomes in this context.

This study investigated the experiences of Brazilian transgender and gender non‐binary youth in school settings, focusing on the following school‐related variables: school climate, absenteeism, and school transfer due to transphobic bullying. The aim was to address the under‐representation of transgender youth in educational research, particularly in Latin America. Additionally, the study provided a descriptive analysis of school connectedness and safety rates among Brazilian transgender youth. Beyond centering a Brazilian sample, this study is novel in (a) examining location‑specific safety (e.g., washrooms, changing rooms), (b) assessing gender‑affirmation support from multiple school actors, and (c) testing whether associations differ by educational context (high school vs post‑secondary).

We frame our work within school climate models, which emphasize the role of relationships, safety, and institutional norms in shaping student engagement and wellbeing (Kearney and Hendron 2016). A positive school climate—characterized by connectedness and perceived safety—has been consistently linked to lower absenteeism and improved academic outcomes (Kearney and Hendron 2016). In parallel, minority stress theory posits that stigma‐related stressors, such as victimization and misgendering, erode mental health and school engagement unless buffered by affirming supports (Meyer 2003; Toomey 2021). For transgender and non‐binary youth, gender‐affirmation practices (e.g., correct name and pronoun use) and supportive peer and teacher relationships can mitigate these stressors and foster belonging (Fantus and Newman 2021; Johns et al. 2021). Accordingly, we focus on three indicators—school connectedness, location‐specific safety, and gender‐affirmation support—as key factors expected to influence absenteeism: low connectedness reduces motivation and belonging; unsafe spaces encourage avoidance; and affirmation may buffer stress, improving attendance.

## 2 Methods

### 2.1 Sample

Participants were invited to take part in a 1 h long questionnaire available online on the Qualtrics platform (Qualtrics, Provo, UT and Seattle, WA). The announcement was displayed for Facebook users who indicated the following characteristics on their profiles: lived in Brazil; were between 13 and 25 years old; and “liked” Facebook content related to the transgender youth movement. A total of 706 adolescents and young adults initiated the survey. The study's focus on transgender and non‐binary youth aged 13 to 25 is justified by the importance of analyzing school climate across a critical educational continuum, from high school through university, an age range consistent with other relevant research on LGBTQ+ youth in Brazil.

To ensure data quality, we removed duplicates (identical IP/metadata), incomplete responses (< 10% items), and discrepant surveys defined a priori as: (a) contradictory answers on the two‑step gender questions; (b) impossible combinations (e.g., indicating “not in school” but completing school‑specific matrices); (c) straight‑lining across long grids; or (d) completion times < 1/3 of the median. Thus, data from 338 participants were excluded from the analyses.

Furthermore, participants who did not identify as transgender or gender non‐binary using the two‐step method questions (sex assigned at birth and current gender identity) were excluded. More precisely, participants answered the question *“*what pronouns do you like to use for yourself? ***”*** choosing between “she/her”, “he/him”, or “something else”. They then described their gender through an open‐ended question *“*what word or words do you use to identify your gender?*”* and answered *“*if you had to pick one of the following, would you say that you are…*”* selecting between the alternatives “male or primarily a boy”, “female or primarily a girl”, and “non‐binary or something other than a male or a female”. Finally, they were asked to disclose their sex assigned at birth. Fifteen participants identified as non‑binary and chose not to disclose sex assigned at birth; they were retained because their gender identity and pronoun responses met inclusion criteria. Fifteen participants identified as non‑binary and chose not to disclose sex assigned at birth; they were retained because their gender identity and pronoun responses met inclusion criteria.

A total of 293 transgender youth were included. Overall, the mean age was 18.71 (SD 2.58) ranging from 13 to 25 years. However, the mean age ranged from 16.74 years for those in high school to 20.43 years for those in post high‐school education. Specifically, the mean age was 16.74 years for high school students, 18.19 years for pre‐university students, 18.50 years for those taking technical courses, 20.43 years for college students, and 19.84 years for individuals not currently in school. For more characteristics of the sample, see Table 1.

**Table 1:** Sample characteristics.

	*n*	%
*Sex at birth (n = 282)*		
Female	202	71.63%
Male	80	28.37%
*Gender identity (n = 291)*		
Transgender girls and women	131	45.02%
Transgender boys and men	65	22.34%
Gender non‐binary	95	32.65%
*Racial background (n = 231)*		
Nonwhite	95	41.13%
White	136	58.87%
*Brazilian region (n = 287)*		
South	88	30.66%
Southeast	120	41.81%
Northeast	13	4.53%
North	41	14.29%
Midwest	25	8.71%
*Location of residence (n = 293)*		
Inside the city	228	77.82%
Outside the city (suburbs or rural areas)	65	22.18%
*Are you currently studying? (n = 281)*		
Yes, high school	103	36.65%
Yes, post‐high school	110	36.14%
Yes, I'm in a pre‐university course	16	5.69%
Yes, I'm in a technical course	20	7.12%
Yes, I'm in college	74	26.33%
No	68	24.20%

### 2.2 Measures

The survey was inspired by the TransYouth CAN! Project (http://transyouthcan.ca/), a cohort study that documents sociodemographic and health‐related characteristics of Canadian transgender and gender non‐binary youth and their parents or caregivers. For further information concerning the cross‐cultural adaptation of the instrument for Brazilian populations, see previous publications (Fontanari et al. 2019; Fontanari, Churchill, et al. 2020a; Fontanari, Vilanova, et al. 2020b). The “White” and “Non‐White” racial categories in Table 1 were derived from the responses to the following categories: White/Caucasian, Brown, Black, Asian, and Indigenous.

#### 2.2.1 Gender Identity

Gender identity was assessed using the two‐step method (Sausa et al. 2009). Based on their self‐reported gender identities, participants were separated into the following groups: transgender girls, transgender boys, and gender non‐binary youth. Transgender girls included youth assigned male at birth who identified as girls, transgender girls, or as *travestis* (a Latin American term that usually refers to people who perform feminine roles without necessarily altering their physical characteristics (Vartabedian 2016). Transgender boys were those assigned females at birth who identified as boys or transgender boys. Finally, gender non‐binary youth include adolescents and young adults who reported having a gender identity outside the binaries of male and female, such as queer, non‐binary, a‐gender, and others.

#### 2.2.2 School Community Support for Gender Affirmation

The survey assessed school community support for gender affirmation across three groups: classmates, general teachers, and extracurricular teachers. Participants responded to items on a Likert‐type scale. For Name Support (“Call by name and pronoun that reflects your true gender identity”), response options were All the time, Sometimes, and Never, plus I didn't ask to. For Identity Support (“Support your gender identity and expression”) and Gender‐Affirmation Support (“Support your gender‐affirming process, including hormone treatment and surgical procedures”), options were Strongly support, Somewhat support, No support, and I didn't ask to. Items were numerically coded as follows: All the time or Strongly support = *3*, Sometimes or Somewhat or low support = *2*, Never or No support = *1*, and I don't have (that person/relative) treated as missing. Composite scores were calculated as means to preserve the original 1–3 scaling and accommodate occasional missingness. Internal consistency was excellent for the 3 domains (Name, Identity and Gender affirmation) (Cronbach's *α* > 0.90), with higher scores indicating greater perceived support. Detailed results for each group (classmates, teachers, and extracurricular teachers) are presented in Table 2.

#### 2.2.3 School Connectedness

This measure is composed of five statements that can be answered with a 5‐point Likert‐type scale. Examples include “I am happy to be at this school*”* or *“*The teachers at this school treat students fairly*”* (Mcneely et al. 2002). The response scale was 1 = *strongly disagree*, 2 = *disagree*, 3 = *neither* disagree nor agree, 4 = *agree*, and 5 = *strongly agree*, with total scores ranging from 5 to 25 (Furlong et al. 2011). In the current study, the Cronbach's *α* was 0.88, higher scores meaning high connectedness.

#### 2.2.4 School Safety

This measure was developed by TransYouth CAN! team (https://transyouthcan.ca/). It is composed of eight questions concerning how often the youth feel safe at school. More precisely, in their classrooms, in the washrooms, in the changing rooms, in the hallways, in the library, in the cafeteria, outside in the school grounds, and on the way to and from school. The response scale was 1 = *never*, 2 = *rarely*, 3 = *sometimes*, 4 = *frequently*, and 5 = *always*. The total scores ranged from 8 to 40. In the current study, the Cronbach's *α* was 0.92, highest scores meaning more safety.

#### 2.2.5 School Disruption Indicators

Participants responded to whether they have been absent from school for more than two consecutive weeks (coded as 0 = *at least once*, 1 = *never*). If they have been absent from school for more than 2 weeks, they were asked to specify how long they were missing for (coded as 1 = *2 to 8 weeks*; and 3 = *more than 8 week*s), and if the absence was related to their gender identity (coded as 0 = *no* and 1 = *yes*). They also responded whether they want to (coded as 0 = *no* and 1 = *yes*) or have already changed schools (coded as 0 = *no* and 1 = *yes*) in response to transphobic bullying.

### 2.3 Statistical Analysis

The data analysis primarily involved descriptive statistics to present the characteristics of the sample and key variables. This included reporting frequencies and percentages for categorical variables like gender identity and school disruption indicators. Additionally, means and standard deviations were provided for continuous variables such as school connectedness and school safety scores, only for those currently studying. Because interpretation of school climate items is ambiguous for those not currently enrolled, we excluded not‐enrolled respondents from all analyses that involve school climate, safety, support, or absenteeism. The analysis was done considering differences by educational context (high school vs. post‐secondary. To examine the relationship between school experiences and absenteeism, s two‐tailed Pearson's correlation was also performed to assess the association between school connectedness, safety, and the variable related to gender support. All analyses were conducted using IBM SPSS 25.0.

## 3 Results

As shown in Table 2, Brazilian transgender youth reported varying levels of support for their gender affirmation, with notable differences by educational context. Overall, students in post‐high school settings reported higher levels of affirmation from classmates and teachers compared to those in high school. For instance, only 19.19% of high school students reported that their teachers “always” use their correct name and pronouns, compared to 43.75% of post‐high school students. Similarly, a higher percentage of high school students reported receiving “no support” for their gender identity from teachers (46.15%) compared to their post‐high school peers (24.%). Support from extracurricular teachers (e.g., coaches) was the lowest across both groups.

**Table 2:** School community support for gender affirmation.

Group			Call by name and pronoun that reflects your true gender identity	Support your gender‐identity and expression	Support your gender‐affirming process, including hormone treatment and surgical procedures
			*n* (%) all‐time	*n* (%) strongly support	*n* (%) strongly support
Classmates	High school		25 (26.04%)	23 (24.73%)	15 (18.75%)
	Post‐High school		38 (38.78%)	33 (35.48%)	21 (24.42%)
			n (%) sometimes	n (%) somewhat support	n (%) somewhat support
	High school		29 (30.21%)	38 (40.86%)	27 (33.75%)
	Post‐High school		31 (31.63%)	44 (47.31%)	42 (48.84%)
			n (%) never	n (%) no support	n (%) no support
	High school		20 (20.83%)	32 (34.41%)	38 (47.50%)
	Post‐High school		12 (12.24%)	16 (17.20%)	23 (26.74%)
			n (%) didn't ask to		
	High school		22 (22.92%)		
	Post‐High school		17 (17.35%)		
			n (%) all‐time	n (%) strongly support	n (%) strongly support
Teachers	High school		19 (19.19%)	18 (19.78%)	10 (12.66%)
	Post‐High school		42 (43.75%)	28 (30.77%)	19 (22.09%)
			n (%) sometimes	n (%) somewhat support	n (%) somewhat support
	High school		21 (21.21%)	31 (34.07%)	28 (35.44%)
	Post‐High school		19 (19.79%)	41 (45.05%)	33 (38.37%)
			n (%) never	n (%) no support	n (%) no support
	High school		26 (26.26%)	42 (46.15%)	41 (51.90%)
	Post‐High school		13 (13.54%)	22 (24.18%)	34 (39.53%)
			n (%) didn't ask to		
	High school		33 (33.33%)		
	Post‐High school		22 (22.92%)		
			n (%) all‐time	n (%) strongly support	n (%) strongly support
Extracurricular teachers (e.g., coaches)	High school		12 (17.39%)	12 (23.08%)	9 (17.31%)
	Post‐High school		23 (38.33%)	12 (24.49%)	7 (14.00%)
			n (%) Sometimes	n (%) somewhat support	n (%) somewhat support
	High school		14 (20.29%)	13 (25.00%)	12 (23.08%)
	Post‐High school		9 (15.00%)	21 (42.86%)	21 (42.00%)
			n (%) Never	n (%) no support	n (%) no support
	High school		21 (30.43%)	27 (51.92%)	31 (59.62%)
	Post‐High school		12 (20.00%)	16 (32.65%)	22 (44.00%)
			n (%) Didn't ask to		
	High school		22 (31.88%)		
	Post‐High school		16 (26.67%)		

Table 3 details the rates of school disruption, again highlighting differences between the two groups. A larger percentage of high school students (48.9%) reported having been absent for more than two consecutive weeks compared to post‐high school students (35.9%). For those who experienced such absences, a majority in both groups indicated the absence was related to their gender identity (65.8% for high school and 70.0% for post‐high school). Over half of the students in both groups reported wanting to change schools due to transphobic bullying, with the rate being slightly higher among high school students (60.2%) than post‐high school students (53.0%).

**Table 3:** School disruption indicators.

	Total^a^		High school		Post‐High school	
	*n*	%	*n*	%	*n*	%
Have you ever been absent from school for more than two consecutive weeks? (*n* = 271)	123	45.39%	45	48.90%	37	35.90%
· · · · · 2–8 weeks	63	51.22%	29	64.40%	20	54.10%
· · · · · > 8 weeks	60	48.78%	16	35.60%	17	45.90%
If you were absent for more than two weeks, was this absence related to your gender identity? (*n* = 104)	67	64.42%	25	65.80%	21	70.00%
Wanted to change schools due to transphobic bullying? (*n* = 268)	149	55.60%	59	60.20%	53	53.00%
Changed schools due to transphobic bullying? (*n* = 279)	49	17.56%	15	15.30%	13	12.30%

^a^ Includes those who are not currently studying.

Table 4 presents the mean scores for school connectedness and safety items, along with independent samples t‐tests comparing students currently enrolled in high school versus post‐high school. The analysis revealed that post‐high school students reported statistically significantly higher feelings of connectedness and safety on most of the items. For example, on the item “I am happy to be at my school,” the mean score for post‐high school students (*M* = 3.09) was significantly higher than for high school students (*M* = 2.66), a difference representing a medium effect size (*t* = −2.89, *p* = 0.004, Cohen's *d* = 0.41). Similarly, the largest difference in perceived safety was found in “hallways and stairwells,” where post‐high school students reported significantly higher safety (*M* = 3.43) than their high school counterparts (*M* = 2.70), also a medium effect size (*t* = −3.62, *p* < 0.001, Cohen's *d* = 0.51). Conversely, while perceived safety was low for both groups in gender‐segregated spaces, the differences between groups in washrooms and changing rooms were not statistically significant. This indicates that these are unsafe environments for transgender and non‐binary youth across all educational levels.

**Table 4:** School connectedness and school safety mean scores.

	High school			Post‐High school			*t*	*p*	Cohen's *d*
	*N*	*M*	SD	*N*	*M*	SD			
*School connectedness*									
I feel close to people at my school.	95	2.45	1.00	101	2.65	0.97	−1.42	0.157	0.20
I feel I am part of my school.	94	2.44	01.03	101	2.73	01.01	−1.98	0.049	0.28
I am happy to be at my school.	95	2.66	01.08	101	03.09	1.00	−2.89	0.004	0.41
The teachers at my school treat me fairly.	90	2.92	0.89	97	3.27	0.84	−2.76	0.006	0.40
I feel safe in my school.	94	2.73	0.93	102	03.09	0.85	−2.82	0.005	0.40
*School Safety (do you feel safe…)*									
In your classroom?	100	03.05	1.43	110	3.64	1.35	−3.07	0.002	0.42
In washrooms?	97	1.97	1.65	109	2.25	1.57	−1.24	0.215	0.17
In changing‐rooms?	96	1.57	1.67	107	1.58	1.68	−0.04	0.966	0.01
In the hallways and stairwells?	99	2.70	1.51	108	3.43	1.38	−3.62	< 0.001	0.51
In the library?	100	3.55	1.53	107	3.88	1.35	−1.64	0.102	0.23
In the cafeteria?	94	2.96	1.72	105	3.43	1.51	−2.04	0.043	0.29
Outside on school grounds?	96	2.34	1.53	107	2.54	1.45	−0.95	0.342	0.13
Getting to/from school?	97	2.14	1.52	107	2.59	1.55	−2.09	0.038	0.29

Focusing only on participants who are currently studying, two independent Welch's t‐tests were conducted to examine differences based on the outcome ‘Have you ever been absent from school for more than two consecutive weeks?’. First, a *t*‐test comparing mean ‘School Connectedness’ scores revealed a statistically significant difference. Students with prolonged absences reported significantly lower levels of school connectedness (*M* = 2.65, SD = 0.90) compared to those who did not have such absences (*M* = 2.93, SD = 0.73), *t*(151.87) = −2.33, *p* = 0.021. The magnitude of this difference was small to medium (Cohen's *d* = −0.35). However, a second *t*‐test comparing mean ‘School Safety’ scores showed no statistically significant difference between the two groups. The group with prolonged absences (*M* = 2.60, SD = 1.32) did not report significantly different school safety scores compared to the group without such absences (*M* = 2.80, SD = 1.17), *t*(159.50) = −1.09, *p* = 0.276.

Finally, a Pearson correlation analysis was conducted separately for each educational context (Table 5). The upper diagonal of the matrix represents correlations for the high school group, while the lower diagonal represents the post‐high school group. For both groups, school safety and school connectedness were positively and significantly correlated, indicating that students who felt safer also felt more connected to their school. Furthermore, all three measures of gender‐affirmation support were strongly and positively correlated with school connectedness in both contexts. The relationships with school safety were also positive, though the strength varied. For example, Gender‐affirmation support was significantly correlated with school safety for high school students (*ρ* = 0.31), but this relationship was weaker and only marginally significant for post‐high school students (*ρ* = 0.24).

**Table 5:** School safety and school connectedness, community support for gender affirmation correlation matrix *ρ* (*n*).

	1	2	3	4	5
1 School safety	—	0.51** (*n* = 100)	0.30** (*n* = 78)	0.36** (*n* = 93)	0.31** (*n* = 80)
2 School connectedness	0.58** (*n* = 108)	—	0.58** (*n* = 79)	0.54** (*n* = 94)	0.61** (*n* = 81)
3 Name Support	0.35** (*n* = 85)	0.31** (*n* = 83)	—	0.72** (*n* = 75)	0.71** (*n* = 68)
4 Identity Support	0.19 (*n* = 96)	0.48** (*n* = 94)	0.50** (*n* = 80)	—	0.84** (*n* = 81)
5 Gender‐affirmation support	0.24* (*n* = 90)	0.56** (*n* = 88)	0.44** (*n* = 78)	0.84** (*n* = 89)	—

*Note:* The correlation values on the upper diagonal represent the associations measured in the “High School” context. The correlation values on the lower diagonal represent the associations measured in the “Post‐High School” context. Significance levels:

** *p* < 0.01 and *p < 0.05.

## 4 Discussion

While the body of research on school climate for transgender youth is expanding, significant gaps remain, particularly in contexts outside of North America (Bear et al. 2016; Franco and Cicillini 2016). This study aimed to address this gap by investigating the impact of school climate, specifically school connectedness and safety, on absenteeism and school transfer due to transphobic bullying among Brazilian transgender and gender non‐binary youth. We found that school connectedness was related to prolonged absenteeism among Brazilian transgender and non‐binary students. Gender‐affirmation support from peers and teachers was associated with higher connectedness and safety, aligning with minority stress theory's emphasis on protective, affirming environments.

Our analysis revealed the differences in school climate between educational contexts. Post‐secondary students reported significantly higher levels of connectedness and safety across multiple domains compared to their high school peers. This finding supports the interpretation that the transition out of the more rigid and often more hostile high school environment into a post‐secondary setting—which may offer more autonomy and diverse social norms—can lead to a tangible improvement in school climate for transgender and non‐binary youth However, it is crucial to note that this improvement was not universal. The lack of a statistically significant difference in perceived safety within gender‐segregated spaces like washrooms and changing rooms is a critical finding. Despite students moving into a generally safer environment, these specific locations remain profoundly unsafe across all educational levels.

Consistent with the study's initial hypotheses and the framework of Minority Stress Theory, which posits that chronic stigma‐related stressors erode mental health and engagement unless buffered by affirming supports (Meyer 2003; Toomey 2021), our results indicate high levels of educational disruption linked to experiences of transphobia. A significant portion of the sample reported disengagement, with 45.4% of all participants experiencing prolonged absences of more than two consecutive weeks, primarily due to their gender identity (64.4%). Moreover, over half (55.6%) had wanted to change schools due to transphobic bullying, and 17.6% had already executed a school transfer for this reason. These figures illustrate the sustained withdrawal that result when institutional environments are hostile and fail to affirm minority identities. We also found that low school connectedness was a factor related to prolonged absenteeism. Students with prolonged absences reported significantly lower mean scores on school connectedness compared to those without such absences. This finding directly supports the hypothesis that a diminished sense of belonging reduces engagement and increases the likelihood of withdrawal, reflecting the proximal stressor of anticipated rejection within the school environment (Johns et al. 2021).

Another central tenet of Minority Stress Theory is that supportive environments can act as protective factors against the chronic stressors faced by marginalized individuals (Meyer 2003). Our analysis validates this mechanism: All three measures of gender‐affirmation support from peers and teachers were strongly and positively correlated with school connectedness in both high school and post‐high school settings (e.g., Name Support and Connectedness: in high school and in post‐high school). This confirms the hypothesis that affirmation practices buffer minority stress, fostering a sense of belonging and resilience, which ultimately improves attendance and reduces the risk of school withdrawal.

From an institutional approach, schools could encourage better attendance among transgender and gender non‐binary students' by focusing on developing a favorable school climate (McGuire et al. 2010a, b). However, Brazilian schools are not known for welcoming young transgender and gender non‐binary students. Episodes of school discrimination, violence, and exclusion, often perpetuated by the school staff, have been reported (Stucky et al. 2020). A poor school climate is an initial step in a lifelong disadvantage trajectory faced by transgender populations, as low levels of education aggravate access to labor (Costa et al. 2020). The consequences are even more far reaching as Brazilian transgender report more difficulties in accessing health services (Cerqueira‐Santos et al. 2010; Costa et al. 2016; Rocon et al. 2018).

The Centers for Disease Control and Prevention (2009) and The Institute for Educational Sciences (2008) recommended enhancing school climate as an evidence‐based approach to reduce dropout rates (Dynarski et al. 2008). Accordingly, the school climate is frequently targeted in school‐based programs aiming to improve students' development and ameliorate behavior issues. Institutional support can assume multiple formats. For example, creating a gay‐straight alliance or similar types of organized activities, supporting educators and other staff to become trained in LGBTQ+ issues, including sex and gender in the curriculum, and implementing comprehensive anti‐bullying policies may improve school climate for transgender students (Feijo et al. 2022; Kosciw et al. 2013).

Considering the repercussions of an unfavorable school climate on the lives of transgender and gender non‐binary youth, Brazilian schools should work on recognizing their transphobic practices, re‐training their staff, and reformulating its curriculum to include sex and gender diversity (Colling 2013; Miskolci 2012). Furthermore, health care providers, as well as school counselors, should engage transgender and gender non‐binary youth and their caregivers about the importance of the school environment, and selecting schools with gay‐straight alliances, supportive staff, inclusive curriculum, and extensive anti‐bullying policies. Additionally, it is relevant to point out that Brazil has the third‐highest rate of school dropout in the world. On average, 40.8% of students do not complete secondary school until the age of 19 (INEP et al. 2019). When considering students from lower SES, the rates are even worse: 91.1% of youth from higher SES complete high school before 19 years old compared to 42.7% of adolescents from lower SES (INEP 2019). In our sample, 24.20% of participants reported not currently studying, a rate that reflects current non‐enrollment rather than a formal, lifetime dropout rate. Caution is needed when comparing this figure to the national rates of non‐completion of secondary school, as our sample is limited by its online nature and may not represent the most vulnerable youth, who often lack internet access.

It must be considered that our results are based on an online survey; only youth with stable internet access were able to fulfill it. In Brazil, less than 50% of low‐SES groups access the internet from their homes (Araujo et al. 2018). Therefore, generalization of these findings must be taken with caution. Besides the selection bias, another limitation is the cross‐sectional nature of our study. Another limitation is related to the operationalization and our variables. Even though the school climate precedes dropout, the same rationally does not apply to absenteeism. Meaning that the trajectory leading to absenteeism is not clearly due to lack of reported school connectedness – some students could have reported a school connectedness and safety that apply to a different school from the one they were absent. Additionally, using an absenteeism threshold of more than 2 consecutive weeks has important implications. This operationalization captures prolonged disengagement rather than short‐term truancy, which may better reflect severe disruptions in educational continuity among transgender and non‐binary youth. However, because this criterion is more stringent than those used in many prior studies (which often assess missed days or weeks without requiring consecutiveness), our prevalence estimates should be interpreted as conservative. This approach likely underestimates less severe absenteeism but highlights cases of sustained withdrawal, which are particularly relevant for understanding risk of academic failure and school transfer. Future research should compare multiple absenteeism metrics to determine whether predictors differ across thresholds and examine whether prolonged absence uniquely predicts long‐term educational outcomes.

Despite these limitations, ultimately, this study highlights that supportive, affirming relationships and location‐specific safety measures are not secondary issues but are crucial, evidence‐based buffers against minority stress, determining whether transgender and non‐binary youth can persist in Brazilian education. Knowing that the school as an institution can positively influence transgender and gender non‐binary students' attendance by investing in a favorable school climate, it is fair to inquire how schools may promote a safer and more inclusive environment.

## Funding

The authors received no specific funding for this work.

## Ethics Statement

The Ethical Committee and Research Commission of [anonymized] approved the project [anonymized]. This decision was made because, for some participants, their gender identity may not have been disclosed to their parents or guardians. This approach was considered and approved by the ethics committee to prioritize the well‐being and safety of all participants.

## Consent

All participants signed an online ethical consent form. Individuals under the legal age of consent in Brazil participated in this study. While the standard protocol requires parental/guardian consent for such participants, the ethics committee specifically authorized a waiver of formal caregiver signatures in certain cases. Requiring formal consent in these instances could have inadvertently revealed this information, potentially representing a situation of family victimization for the young person.

## Conflicts of Interest

The authors declare no conflicts of interest.

## Data Availability

The data that support the findings of this study are available on request from the corresponding author. The data are not publicly available due to privacy or ethical restrictions.
